# “Does the Response to Morning Medication Predict the ADL-Level of the Day in Parkinson's Disease?”

**DOI:** 10.1155/2020/7140984

**Published:** 2020-07-27

**Authors:** Trine Hørmann Thomsen, Troels Wesenberg Kjær, Lene Bastrup Jørgensen, Anita Haahr, Kristian Winge

**Affiliations:** ^1^Department of Clinical Neurophysiology and Neurology, Zealand University Hospital, Roskilde, Denmark; ^2^Department of Clinical Medicine, University of Copenhagen, Copenhagen, Denmark; ^3^Center of Planned Surgery, Regional Hospital, Silkeborg, Denmark; ^4^Department of Clinical Medicine, University of Aarhus, Aarhus, Denmark; ^5^Center for Health Promotion and Rehabilitation, VIA University College, Aarhus, Denmark; ^6^Novo Nordisk Foundation, Hellerup, Denmark

## Abstract

**Background:**

Individuals with Parkinson's Disease (PD) have bradykinesia during mobility tasks in the morning before intake of dopaminergic treatment and have difficulties managing Activities of Daily Living (ADLs). Early morning off (EMO) refers to off-states in the morning where the severity of bradykinesia is increased and causes a decrease in mobility related to wearing off of effects of medication. Measurements from devices capable of continuously recording motor symptoms may provide insight into the patient's response to medication and possible impact on ADLs.

**Objectives:**

To test whether poor or slow response to medication in the morning predicts the overall ADL-level and to assess the association between change in bradykinesia score (BKS) and the risk of having disabilities within three selected ADL-items.

**Methods:**

In this cross-sectional study, the sample consists of 34 patients with light to moderate PD. Data collection encompasses measurements from the Parkinson KinetiGraph, and the ADL-limitations are assessed by the Movement Disorder Society Unified Parkinson Disease Rating Scale (MDS-UPDRS) Part II.

**Results:**

The association between UPDRS- II and BKS from the algorithm was −0.082 (*p* < 0.01), 95% CL:−0.113; −0.042). The individuals experienced disabilities in performing “Speech” (*p*=0.004) and “Doing hobbies” (*p*=0.038) when being slow or poor responders to dopaminergic therapy. The PD patients' L-dopa equivalent dose seems to be a strong predictor of the ADL-level in the morning.

**Conclusion:**

Slow response to the medication dosages in the morning is correlated with disabilities in the overall ADL-level in PD. The combination of PD-drugs and precise, timely dosages must be considered in the improvement of the ADL-level in PD patients.

## 1. Introduction

Parkinson's Disease (PD) is a progressive, neurodegenerative disease. Patients with PD exhibit heterogeneous clinical phenotypes, and there are a large variability in the symptoms [1] and, hence, courses of disease. Patients with PD are challenged by the classical triad of motor symptoms, Bradykinesia, rigidity, and tremor, and in moderate to severe stage, impairment in gait and balance [2]. Bradykinesia is defined as slowness of initiation of voluntary movement with progressive reduction in speed (ibid). Bradykinesia correlates well with the dopaminergic deficiency. PD patients are also challenged by nonmotor symptoms as cognitive and autonomic symptoms as well as neuropsychiatric difficulties [1]. Thus, living with PD influences the physical, mental, and social health of patients, and there is an increasing need to understand the impact of PD symptomatology on the ability to perform activities of daily living (ADL) to be able to provide individualized targeted treatment. Patients with mild to moderate PD may only have minor impairments regarding ADLs, and by quantifying their ADL-level, it is possible that targeting will be more efficient prospectively within this group [3].

ADLs are characterized as the activities we perform every day such as getting dressed, taking a shower, and cooking. [4]. The ability to perform ADL's depends on overall mobility, cognitive capability, and social support, among others and is highly associated with health-related quality of life (QOL) [5, 6]. In PD, the ADL-level also depends on response to medication and is a dominant factor in managing daily life with PD. Fluctuations in symptoms due to complex response to medication cause pendulum between periods in which PD patients are able to move smoothly for some hours (On state) and periods with increase of motor symptoms (Off-state) [1]. Early Morning Off-episodes (EMOs) refer to periods with off-states in the morning where patients present with poor motor function due to an increase in the severity of bradykinesia [2]. It is currently considered that off-periods in early and moderate stages are related to the wearing off of effects of dopaminergic medication and that they can be relieved by keeping plasma levels of medication stable [7]. EMOs occur when the effects of PD medicine wear off during the night, as levels of medication drop until the first dose of dopaminergic therapy is due. Experiencing EMOs can be a frustrating complication to PD, as the patients show difficulties managing ADL-routines such as getting out of bed and getting dressed [8].

The irregular nature of motor manifestations requires observations from within the patient's environment to obtain a realistic picture of the management of the symptoms and execution of ADLs [9, 10]. Therefore, objective measurements from wearable devices capable of continuously recording motor symptoms and fluctuations may help provide insight into PD patient's everyday life rather than data from, e.g., self-reported diaries alone, which is traditionally used. To make therapy adjustments, reduce impact of motor symptoms and fluctuations, and thereby improve the ADL-level, detailed information of these areas is important [10, 11]. Robust accuracy and validity metrics for some features have been reported [12–14] and may improve the response to treatment. Until now, objective assessment of response to medication during ADLs in the home has not been extensively examined. An accurate report of PD motor states may enable health care professionals to personalize medication intake and, thus, improve response to treatment.

In assessing the disease severity in clinical settings, the Movement Disorder's Society's Unified Parkinson's Disease Rating Scale (MDS-UPDRS) is traditionally used. Part II of the MDS-UPDRS is related to the evaluation of specific motor aspects of experiences of daily living and can provide a status of the ability to perform ADLs, thus, impacting everyday life and activities [15]. To our knowledge, the MDS-UPDRS Part II as a major outcome measure in clinical research in terms of the responsiveness to medication has not been investigated in a prospective designed study.

In this study, we aim to detect change in bradykinesia state after taking the first morning dosage of dopaminergic treatment and extract features from accelerometer measurements to test whether slow response to morning medication predicts the overall ADL-level in patients with PD. At the same time, we want to assess the correlation between change in bradykinesia score, the medicine profile, and the risk of having disabilities within three selected items in the MDS-UPDRS Part II.

## 2. Materials and Methods

### 2.1. Design

This cross-sectional study constitutes the first phase of a mixed methods study.

### 2.2. Participants

Thirty-four patients with PD were included. The participants had mild to moderate PD (Hoehn and Yahr scale > 1 < 4), fulfilling the Movement Disorder Society diagnostic criteria [16], nondemented based on a cut-off score >26 in The Montreal Cognitive Assessment scale (MoCA) [17], age of 50–75 years, and an illness duration of 3–7 years. The limit of numbers of PD-drugs was set to a minimum of 1 and a maximum of 4. Exclusion criteria were patients with advanced treatments, dementia, and severity in comorbidity, and a high degree of comorbidity (cut-off < 5, expressed in the Charlsson Comorbidity Index) [18].

The participants were recruited consecutively from a Movement Disorders clinic located on Zealand University Hospital, Denmark, and from a recruitment notice in the magazine for members of the Danish Parkinson Association. The participants were assessed clinically for the presence and nature of their motor symptoms before they were included in the study. The goal was to recruit a cohort of 30–35 patients. There were no previous studies that allowed for more detailed power calculation. Therefore, the sample size was based on previous experience in similar studies, but with the MDS-UPDRS Part III as the major outcome [19, 20].

### 2.3. Assessment of Severity in Bradykinesia and Response to Medication

The patients wore an accelerometer, The Parkinson KinetiGraph (PKG; Global Kinetics Corporation) [21] is used for a period of 6 days. They were not suggested to or prevented from doing any specific tasks. The device was worn on the wrist of the most severely affected arm. The PKG was programmed to vibrate to alert the patient when a dose of dopaminergic therapy was due.

The PKG has been demonstrated to be an effective tool for quantifying bradykinesia as well as for capturing the effect of therapeutic interventions [22, 23]. The PKG system is an algorithm-based movement recording platform, which continuously measures movement accelerations and analyzes the spectral power of the low frequencies of the accelerometer data, and thereby causes a measurement of the movement patterns of PD patients in their homes or in clinical settings [23, 24]. The algorithm has been tested on both PD patients and age-matched controls, and the validation data showed that it is able to measure changes in the function state in response to levodopa dosages [23].

The PKG detected the presence and severity of bradykinesia as a measure of response to the intake of dopaminergic treatment. All movements were recorded and processed through the algorithm that determined the bradykinesia score (BKS). The BKS is defined as the mean spectral power surrounding the maximum acceleration within a 2-minute epoch [14, 21]. All BKS of epochs on every 2 minutes in the period 05–11.00 am were extracted from raw data. That is, for every participant, an output of 180 BKS is distributed over the first 6 hours, and these calculations were furthermore divided into T1 and T2. BKS-change is the mean change between T1 and T2. The time period was chosen since 82% of the participants experienced EMO-periods in this time lap.

**Figure 1:**
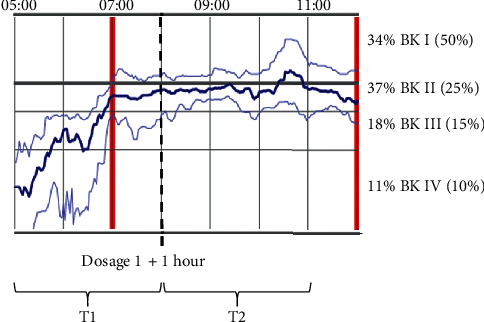
Example of average BKS-level and fluctuations (one patient) during the days, including the two time periods, T1 and T2.

To assess the change in BKS after the first medicine intake in the morning, we divided the measurements in two time periods: (1) predose effect from 05 am to time of first intake plus one hour (expected maximum peak, T1) and (2) postdose effect, from one hour after intake to 11.00 am (T2) (Figure 1). The thick red line indicates time for therapy intake. All participants were instructed to follow the current national guidelines regarding mealtimes half an hour before or one hour after intake of PD-medication due to malabsorption.

The highlighted blue line is the median value for the bradykinesia score (calculated every 2 minutes), for all the days, and is mathematically smoothed over 30 minutes. The 25th and 75th percentiles for the BKS appear as thinner blue lines on each side of the highlighted blue line. The downward axis of the *y*-axis corresponds to an increase in bradykinesia (greatest severity in quartiles III + IV). Thus, a significant change in the BKS (high score) indicates a decrease in bradykinesia severity after intake of medication, and the BKS of epochs will be placed in quartiles I + II.

In previous studies, patients with PD often report “off-state” in periods where the BKS fluctuates between quartiles III and IV [25, 26]. We were only interested in quartiles BK III and BK IV as they correspond to the most severe level of BKS and indicate off-state periods, thus poor or slow response to medication.

### 2.4. Motor Aspects of Experiences of Daily Living (MDS-UPDRS- II)

We used the MDS-UPDRS-II as the main outcome in quantifying the ADL-level. Part II is a series of questions regarding task performance during ADL's and provides a score from 0 to 52 points [15]. The MDS-UPDRS is regarded as the “gold standard” of assessment for individuals with PD [19, 27]. The MDS-UPDRS-II-score was used to compare and detect a possible correlation between the objective BKS and the participant's subjective assessment of the symptoms impact on the ADL-level.

Three of the 13 items were selected in order to examine the relationship between these and the change in BKS. The designation of the 3 items was based on existing literature in which items are known to be some of the most dominant factors in daily life with PD: (1) “Speech,” (2) “Performing hobbies and other activities,” and (3) “Walking and balance” [28, 29], but also from the patients' verbalized limitations in daily life with PD. Further, the effect of dopaminergic therapy seems to be positively related to motor speech disorders in the early phases of PD and appears also to be related to the dopaminergic responsiveness of bradykinesia [30].

### 2.5. Statistical Analysis

Statistical analyses were conducted with the *R* Studio software package (version 1.2.1335).

Analysis was made by linear regression analysis as we wanted to explore the relationship between independent predictors (change in BKS distributed on 6 days) with the dependent outcome of interest, the MDS-UPDRS-II-score. This was controlled for repeated measurements in participants to avoid positive correlation and modify the effect of each participant.

We also used an analysis of variance (ANOVA) and a following Tukey-test to test the day-by-day variance in BKS. The analysis showed no variance within the 6 days (*p* > 0,89). Therefore, the registrations for all the days were merged. Furthermore, a possible difference in mean BKS between participants using one, two, three, or four dopaminergic drugs was tested by using a two-side ANOVA. The test showed no significant difference between number of PD-drugs and the total score in MDS-UPDRS-II (*p* < 0,076), though the individuals differed in the combination of their dopaminergic treatment in terms of total dose of levodopa and agonist-treatment. Therefore, the levodopa equivalent dose (LED) and the agonist eq-dose for each participant were calculated to express the dose intensity of the anti-Parkinsonian drugs [31]. We used an ANCOVA to adjust for the eq-doses between the two groups compared to the UPDRS-score as the independent variable.

Data obtained in the PKG-measurements of the 34 PD patients were initially analyzed separately, and BKS values were divided into the two time periods, T1 and T2. We calculated both mean BKS in T1 and T2 and the absolute differences between the means per individual. The absolute difference from the BKS of each participant after the first medication dosage of the day + one hour was compared to the overall UPDRS-II-score as a continuous numeric variable. A regression analysis was conducted between the measurements (BKS outputs) and the total MDS-UPDRS-II-score to obtain a possible correlation between the variables. We considered *p* < 0.05 as statistically significant.

Additionally, each of the 13 items of the MDS-UPDRS Part II was divided dichotomously into two groups “no disabilities” and “disabilities.” The items were to be answered within five categories; normal, slight, mild, moderate, and severe. In our analysis, we divided answers given as “normal” (score = 0) as equal to “no disabilities.” The rest of the categories were converted into the group “disabilities” (score ≥ 1). To predict the probability to be in the group “disabilities” within the 3 selected items, based on the change in BKS as the predictor (mean BKS-change = 28. BKS-change <28 = “good responder”, >28 = “poor responder”), we performed the analysis using a logistic regression. Thus, with the logit function, we modelled the binary outcome “Disabilities” and “Nondisabilities”.

## 3. Results

**Figure 2:**
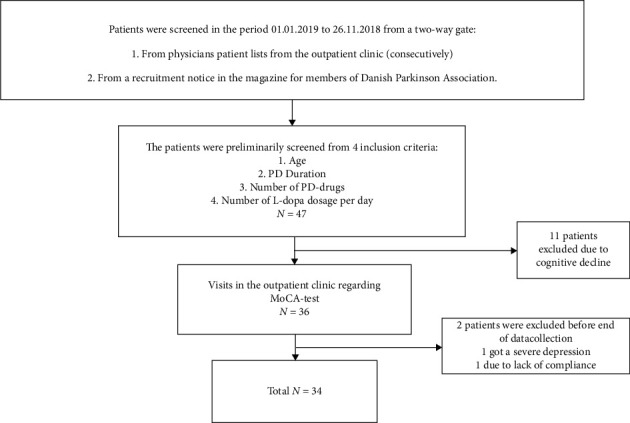
Recruitment and inclusion overview.

Out of 47 initially recruited persons, 11 persons were excluded due to a low score in the MoCA-test. Two persons dropped out of the study. A total of 34 patients with PD fulfilled the required criteria, and their data were completed (Figure 2).

All patients received drug combinations of levodopa, dopamine receptor agonists, MAO‐B, and/or COMT inhibitors. Ninety-four percent of the patients received 2-3 different drugs (Table 1) with a comparable profile of dopaminergic therapy due to T½. Only one of the patients was treated with anti-gastric ulcer medicine, and two patients received antithrombotic therapy. In general, a low mean comorbidity score was expressed. Before the PKG-measurements were recorded, all the patients' motor condition was clinical and subjectively rated as “On” after intake of medication (MDS-UPDRS Part III, data not reported).

**Figure 3:**
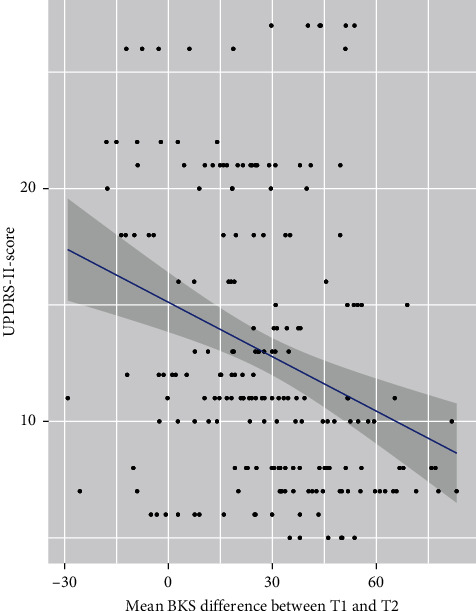
Scatter plot of the differences in BKS against UPDRS Part II total score with embedded interval of confidence.

The association between MDS-UPDRS-II and BKS-outputs from the algorithm was −0.082 (*p* < 0.00002, CL: −0.113; −0.042). The significant differences demonstrate that changes in BKS are associated with changes in the MDS-UPDRS-II score. The coefficients suggest that, for each one-point increase in BKS change, it leads to a decrease in MDS-UPDRS II-score by approximately 0.082 × 100 = 8.2 point (100% expansion), in average (Figure 3).

Hence, a low score in the scale indicates less ADL-impairments. Therefore, the detection of change in BKS in EMO-periods enables us to test whether poor response to medication in the morning predicts the overall ADL-level in patients with PD. A greater difference in the mean BKS-change between T1 and T2 will improve the function state, and, thus, the ability to perform ADLs. The variance of the residuals is due to the biological variation in this type of measurements.

The calculation of eq-dose for each of the participants showed a significant difference between the two groups “good responders” (group 1) and “poor responders” (group 2) (Table 2). Group 2 has a significantly higher total eq-dose and LED than Group 1, which is associated with a smaller change in BKS (poor/slow response) and more severe ADL-impairments (high score in UPDRS-II). This was not associated with PD-duration.

The ANCOVA showed a significant difference in the UPDRS-score between the two groups when adjusting for differences in total eq-dose (*p*=0.0039, 95% CL: 0.0002; 0.0078). The slope was rather small (0.0004), but there were no interactions (*p*=0,53) meaning that the effect of the eq-dose on the UPDRS-score is the same in both groups. When dividing the total eq-dose in LED and agonist eq-dose, it showed that the LED is a strong, independent predictor of the ADL-level (*p*=<0.01, 95% CL: 0.0076; 0.0154) as a higher LED (*x*-axis) is associated with a poor response to morning medication and, thus, an increase in UPDRS-II-score (*y*-axis) (Figure 4 in supplementals).

The association between the change in BKS and the three specific items from the UPDRS-II (1) “Speech,” (2) “Hobbies and other activities,” and (3) “Walking and balance” was analyzed using logistic regression analysis. The probability of having disabilities within each of the 3 items is demonstrated in the model below (Table 3).

Being in the group “poor responder” is associated with an OR = 0.95 (95% CL 0.89; 0.95) of having disabilities within the item “Hobbies and other activities” (Table 3). This indicates that, for each one-point increase in BKS-change, the probability for being in the “disability” group will decrease by 5%. OR in “walking and balance” was insignificant, indicating that a poor/slow change in BKS is not associated with disabilities within this item.

## 4. Discussion

To our knowledge, this is the first study that examines the relationship between response to medication in the morning and the ADL-level for individuals with PD in a prospective design. There is a significant correlation between slow response to medication in the morning and the overall ADL-limitations, which is important knowledge in a clinical perspective, though the participants may have underrated their disabilities, as self-assessed underrating of disability is associated with earlier stages of PD, living with family, and high cognitive ability [32], which characterize our cohort. Specific knowledge about the individual patient's response to medication as an identification of change in BKS may be useful in the predicting, treatment, and better understanding of the disease progression. Patients with more severe and rapidly progressing PD may have a stronger correlation between mean BKS and a decrease in overall MDS-UPDRS- II-score after intake of dopaminergic therapy [33]. However, a precise organization of timely dosages of medicine seems to stress the importance related to the execution of ADLs throughout the day.

The importance of timely dosages is also highlighted in the comparison of the eq-doses and the association with BKS-changes and ADL-impairments (UPDRS-II). Remarkably, the group with poor response to morning medication presented a higher total eq-dose and higher LED than the group with good response even though the opposite was hypothesized. This may indicate that the poor response to dopaminergic treatment is being compensated by an increase of the L-dopa treatment. Therefore, this group will probably experience “off-states” in the morning, as a high LED dose will cause a decrease in serum L-dopa due to the lower T½ of L-dopa in comparison to dopamine agonists [34]. On the contrary, it seems that individuals with only agonist-treatment are more covered in terms of eq-dose and also have less ADL-impairments. This aspect must be considered in the treatment of the individual PD patient. Patients with doses of L-dopa higher than 400 mg per day may response positive if the morning dose was increased, and the dopamine agonist is taken mid-day rather than morning in order to increase the early morning effects for less ADL-impairments in the morning. However, further studies are needed to describe the impact of these suggestions.

The individuals with poor responsiveness to dopaminergic therapy based on their change in BKS did not experience or report disabilities in the item “walking and balance.” This result cannot be explained on the basis of the profiles of the “good” versus “poor responders.” There were no significant differences in comorbidity, medicine profile (e.g., anti-gastric ulcer medicine), and constipation due to ventricle emptying and thus effect of the dopaminergic treatment between the two groups. Traditionally, gait has been one of the most dominant features when measuring, e.g., QOL in life with PD [3, 35]. However, Espay et al. showed that PD patients' priorities and sources of disability often arise from nonmotor deficits (e.g., apathy, sleep disturbances, and orthostatic hypotension), and not necessarily from motor symptoms or fluctuations [36]. Our analysis revealed significant associations between the response to medication dosages in the morning and the items “speech” and “hobbies and other activities” in MDS-UPDRS-II. The results may indicate that lack of capacity to maintain social activities and to interact with other people is one of the most bothersome disabilities in life with PD, when being a poor responder to dopaminergic treatment. This suggests the need for incorporating and identifying the most bothersome ADL-impairments, both motor and nonmotor, when identifying poor response to medication.

PKG-measurements reveal how patients function at home in their natural environment in a way that other clinical measurements cannot capture. Hence, the PKG can be useful in the quantification of the ADL-level with a fusion of clinical assessments. Evaluation of the patient's symptoms in clinical settings may be influenced by the patient's wish to make a convincing impression during a short-term examination as well as the physician's subjectivity. Therefore, it may not result in sufficient insight in patients' impairments [37]. By identifying EMO's in relation to BKS-changes, the PKG-measurements can be used to provide tailored feedback to individual patients and possibly predict the responsiveness to treatment.

Some limitations of this study need to be discussed. First, the measurement of the ADL-level was based on MDS-UPDRS-II. It is a highly validated scale, which is used in many similar studies, but it has a subjective nature and is self-reported, and we have to consider some degree of over- and underestimation, which may be viewed as skewing results. Further, we dichotomized the ratings into two groups (disabilities/nondisabilities), which may have simplified data. Thus, distinguishing between “slight” and “moderate” is based on a self-reported evaluation and can differ from person to person even though they have the same degree of severity. Last, it would have been advantageous to access the actual mathematical algorithms for the PKG to accurately determine on- and off-states, but due to proprietary, The Global Kinetics could not release the data.

It should also be emphasized that the cross-sectional nature of the data limits the ability to identify effect and changes over time. Furthermore, we investigated a relatively, mildly to moderately affected PD-cohort, and so the generalizability to later stage PD patients who typically have more disabling fluctuations, and response to medication remains to be addressed.

## 5. Conclusion

Our findings show that slow response to the medication dosages in the morning is associated with disabilities in the overall ADL-level in PD and seems to be connected to non-motor ADL-disabilities. Inclusion of objective measurements from wearable devices obtained in natural environment may support and improve treatment of PD by providing comprehensive symptom data that can enable clinicians to include the response to the dopaminergic treatment in the morning. These should be combined with clinical assessments to capture the complex interplay between response to medication, EMOs, and ADL-limitations in PD in the individualization of the treatment. The combination of PD-drugs and precise, timely dosages must be considered in the improvement of the ADL-level. Consequently, the treatment approach will be tailored to each patient's specific needs and disabilities as an individualized disease “fingerprint.”

## Figures and Tables

**Table 1:** Baseline demographics and characteristics for the participants.

	Mean	Min.–Max.
Age	66,4	53–74
Weight (kg)	79,9	53–141
Height (cm)	174,0	1,57–1,89
Years of disease duration	5,0	3–7
Hoehn&Yahr	2,2	2–3
MoCa-score	27,6	26–30
Comorbidity	1,4	1–5

	*N*	%

Women	18	53,0
Men	16	47,0
Cohabiting	25	73,0
Living alone	9	27,0
Primary school [7–12]	3	9,0
Higher education	25	73,0
Vocational	6	18,0
Number of PD-drugs [1]	2	6
Number of PD-drugs [2, 3]	32	94,0
Number of PD-dosages [1, 2]	10	29,4
Number of PD-dosages [3, 4]	24	70,6

	*Mean*	*Min*.-*Max*.
MDS-UPDRS part II score	12,92 (Median = 9)	5–27

*# Item* 1, 8 & 12 (3 *selected items)*	*Median*	*Min*.-*Max*.
Speech	2,0	0–3
Hobbies and other activities	1,0	0–2
Walking and balance	1,0	0–3

**Table 2:** Overview of the two groups with the EQ-doses included.

Groups^*∗*^	Total EQ-dose mean (sd)	EQ-Levodopa mean (sd)	EQ-agonist mean (sd)	UPDRS-score mean (sd)	BKS-change mean (sd)	Years of PD-duration mean (sd)
Gr.1 + 2	607,5 (207, 3)	328,8 (198, 8)	255,1 (187, 2)	10,5 (4, 7)	27,9^*∗∗∗*^ (24,5)	5,0 (2, 3)
Gr. 1	500,1 (142, 4)	264,9 (178, 4)	173,6 (131, 2)	7,4^*∗∗*^ (2, 3)	−43,7 (28,4)	4,7 (1, 6)
Gr. 2	692,3 (163, 5)	359,6 (171, 6)	162,2 (106, 3)	13 (5, 1)	−15,4 (3, 8)	5,3 (2, 2)
*p*-values	0,006	0,003	0,761	0,0002	0,0052	0,063

^*∗*^Group 1 = Good responders. Group 2 = Poor responders. ^*∗∗*^Low score in UPDRS-II indicates less ADL-impairments. ^*∗∗∗*^Cut-off value between poor and good responders.

**Table 3:** Results of logistic regression analysis of the three selected items.

#Item 1, 8, 12	OR (95% CL)	*p*-value
Hobbies and other activities	0.95 (0.89; 0.95)	0.038
Speech	0.96 (0.89; 0.95)	0.004
Walking and balance	0.67 (0.35; 9.90)	0.265

## Data Availability

The accelerometer data used to support the findings of this study were supplied by Global Kinetics Corporation under license and so cannot be made freely available.

## Supplementary Materials

Supplementary Materials Scatterplot of the association between the UPDRS-score and LED for both groups showing that LED has the same effect in both groups, but poor responders (group 2) score higher in the UPDRS-II (high score = increased degree of impairments).
