# Supplementary figures and images for: “Does the Response to Morning Medication Predict the ADL-Level of the Day in Parkinson's Disease?”
Source: Parkinsons Dis. 2020 Jul 27;2020:7140984. doi: 10.1155/2020/7140984 (PMC7403929; doi:10.1155/2020/7140984)

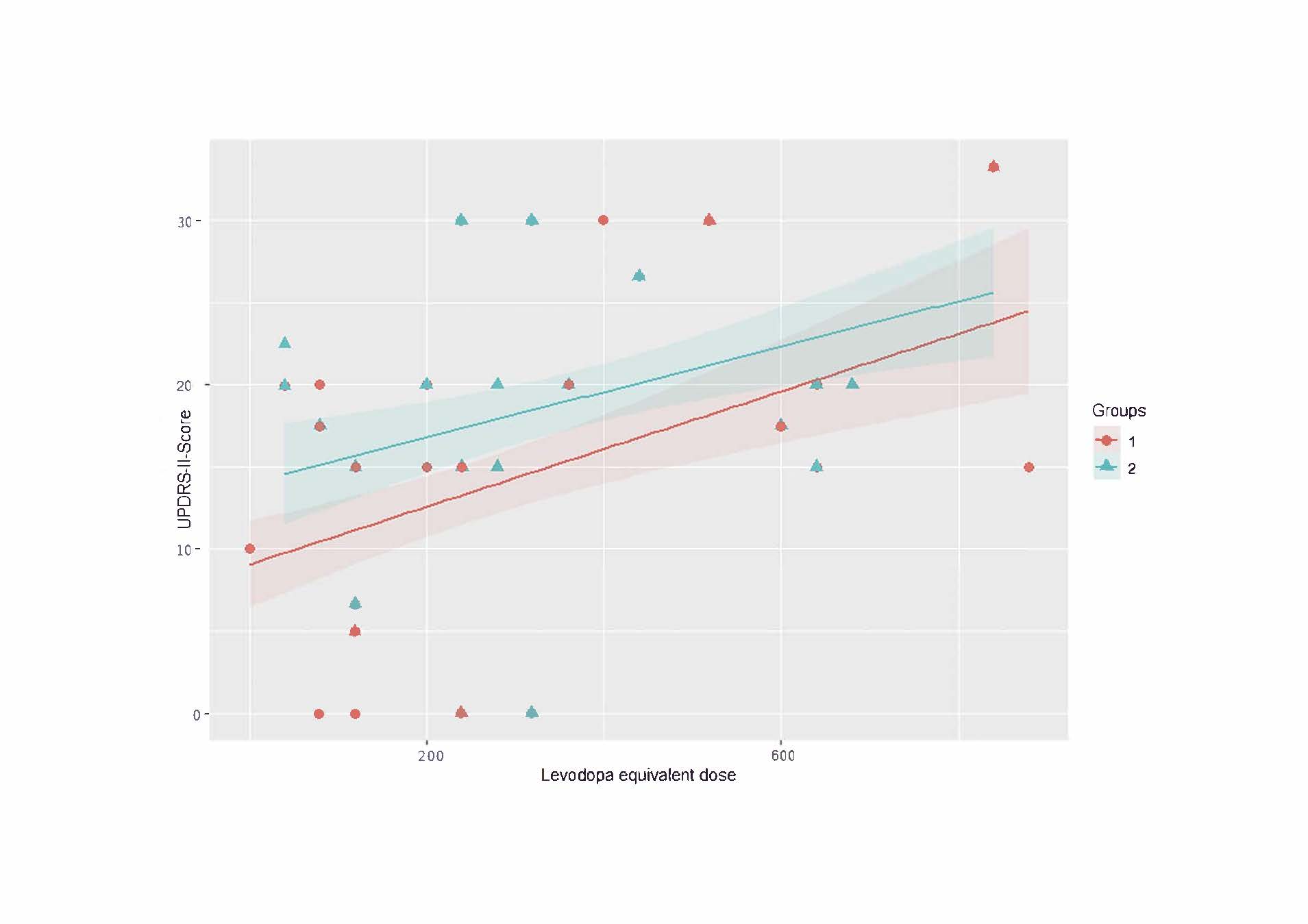

Supplement: Supplementary Materials — Scatterplot of the association between the UPDRS-score and LED for both groups showing that LED has the same effect in both groups, but poor responders (group 2) score higher in the UPDRS-II (high score = increased degree of impairments). [file 7140984.f1.jpg]
